# Cerebral Cryptococcomas: A Systematic Scoping Review of Available Evidence to Facilitate Diagnosis and Treatment

**DOI:** 10.3390/pathogens11020205

**Published:** 2022-02-03

**Authors:** Daniel B. Chastain, Amy Rao, Armaan Yaseyyedi, Andrés F. Henao-Martínez, Thomas Borges, Carlos Franco-Paredes

**Affiliations:** 1 Department of Clinical and Administrative Pharmacy, University of Georgia College of Pharmacy, Albany, GA 31701, USA; 2 School of Medicine, University of Colorado Denver, Aurora, CO 80045, USA; amy.rao@cuanschutz.edu (A.R.); armaan.yaseyyedi@cuanschutz.edu (A.Y.); 3 Division of Infectious Diseases, University of Colorado, Anschutz Medical Campus, Aurora, CO 80045, USA; andres.henaomartinez@cuanschutz.edu (A.F.H.-M.); carlos.franco.paredes@cuanschutz.edu (C.F.-P.); 4 Department of Radiology, University of Colorado, Anschutz Medical Campus, Aurora, CO 80045, USA; thomas.borges@cuanschutz.edu; 5 Hospital Infantil de México Federico Gómez, Mexico City 06720, Mexico

**Keywords:** *Cryptococcus neoformans*, *Cryptococcus gattii*, cryptococcosis, cryptococcoma, cerebral cryptococcosis, neurocryptococcosis, intracranial cryptococcosis, fungi

## Abstract

Background: Recommendations for managing patients with cerebral cryptococcomas are scarce across multiple clinical guidelines. Due to the deficiency of high-quality data coupled with an increasing number of at-risk patients, the purpose of this review is to describe the demographic characteristics, causative pathogen, intracranial imaging, surgical and/or pharmacological interventions, as well as outcomes of patients with cerebral cryptococcomas to improve recognition and management. Methods: We conducted a scoping review in accordance with the PRISMA guidelines using PubMed and Web of Science. Reports were included if the following details were presented: (1) site of infection; (2) treatment details which at least include the specific antifungal therapy administered, if applicable; and (3) patient outcome. Results: A total of 40 records representing 47 individual patients were included, of which the median age was 48.5 years, 75% were male, and 60% reported a significant past medical, surgical, or social history. *C*. *neoformans* was isolated more often than *C*. *gattii* (74% vs. 26%, respectively). Patients most often presented with headache, altered mental status and/or confusion, and vomiting occurring over a median of 30 days; though few were noted to have significant findings on physical examination. More than 50% of patients had a single cerebral cryptococcoma lesion, whereas perilesional edema was present in 73% of cases. Surgical intervention occurred in 49% of patients. An amphotericin B-based formulation was administered as “induction” therapy to 91% of patients, but combined with flucytosine or fluconazole in only 58%, for an overall median of 42 days. Fifty two percent of patients received “maintenance” therapy for a median of 126 days, in which fluconazole was most often used. Corticosteroids were administered to approximately 30% of patients for a median of 31.5 days. Overall, mortality was 34%. Conclusion: Based on our findings, management should include antifungal therapy for a minimum of 6 months with considerations for concomitant corticosteroids in the setting of perilesional edema, as well as surgical intervention. Emphasis should be placed on providing well-documented treatment details in future case reports and series to allow for the development of more concise evidence-based recommendations.

## 1. Background

Despite a decreasing incidence among persons living with HIV due to antiretroviral therapy (ART), rates of cryptococcosis have been increasing in patients who have undergone solid organ transplant or hematopoietic cell transplant [1]. In addition, cryptococcosis is occurring more frequently in non-HIV-infected, non-transplant patients, including those with malignancies, autoimmune diseases, diabetes mellitus, cirrhosis, as well as those receiving immunosuppressive medications [2,3,4,5,6,7,8]. Though most patients have one or more risk factors for cryptococcosis, approximately 30% do not [8]. 

Disseminated *Cryptococcus* spp. infection most often results in meningoencephalitis (CM), but may lead to development of a focal parenchymal brain mass known as cerebral cryptococcoma [9]. After accumulating and infecting the perivascular and subarachnoid spaces, *Cryptococcus* spp. may then invade the brain parenchyma [10,11]. Though the exact mechanism and overall incidence are unclear, cases of cerebral cryptococcomas have been reported in both immunocompetent and immunodeficient patients [12]. Recommendations for managing patients with cerebral cryptococcomas are scarce across multiple clinical guidelines [13,14,15]. Most data comprise a select few patients with cerebral cryptococcomas enrolled in larger studies of patients with CM, studies conducted prior to introduction of azole antifungals and ART, in addition to case reports and expert opinion. Due to the deficiency of high-quality data coupled with an increasing number of at-risk patients, the purpose of this review is to describe the demographic characteristics, causative pathogen, intracranial imaging, surgical and/or pharmacological interventions, as well as outcomes of patients with cerebral cryptococcomas to improve recognition and management.

## 2. Methods

### 2.1. Search Strategy

This scoping review was conducted in accordance with the Preferred Reporting Items for Systematic Reviews and Meta-analyses (PRISMA) guidelines [16]. To identify and select cases for inclusion [17], a systematic literature search using PubMed and Web of Science was performed from 1 January 2010 through 25 May 2021. The literature search only included articles published since 2010, as the practice guidelines for the management of cryptococcal disease were last updated by the Infectious Diseases Society of America using data through December 2009 [14]. The following search terms were used: “cryptococcoma”, “neurocryptococcosis”, “intracranial cryptococcosis”, and “cerebral cryptococcosis”. References within articles of interest were scanned to capture additional sources.

### 2.2. Inclusion and Exclusion Criteria

English language publications were considered, and results were limited to studies, case reports, and case series involving human subjects at least 18 years of age with evidence of cerebral cryptococcoma. To be included, the report must have provided the following details: (1) site of infection; (2) treatment details which at least include the specific antifungal therapy administered, if applicable; and (3) patient outcome. Articles that did not provide disease-specific treatment details or outcomes, as well as conference abstracts, editorials, and review articles were excluded. 

### 2.3. Data Extraction Process

Articles were screened by title and abstract for possible inclusion by three reviewers (DBC, AR, and AY). Full text was then sought for articles that met the inclusion criteria. Data abstracted included demographic characteristics; medical, surgical, and social history; causative pathogens; clinical manifestations; site and description of lesion(s); treatment details, including the specific antifungal therapy, dose, route, and treatment duration; and surgical and/or adjunctive therapies, if applicable, as well as outcome.

## 3. Results

A total of 269 records were identified. After removing duplicates and exclusions, 74 full-text records were screened for eligibility, of which 49 were excluded (Supplementary Figure S1) [16]. An additional 15 records, some of which were published prior to 2010, were identified from references within articles of interest for a total of 40 records [9,18,19,20,21,22,23,24,25,26,27,28,29,30,31,32,33,34,35,36,37,38,39,40,41,42,43,44,45,46,47,48,49,50,51,52,53,54,55,56] representing 47 individual patients included in the systematic scoping review (Table 1).

### 3.1. Demographic Characteristics

Patient ages ranged from 19 to 75 years (median 48.5 years), and 75% (n = 35) were male. Though 40% (n = 19) of the patients did not report a past medical, surgical, or social history, of the 60% (n = 28) who did, 36% (n = 10) were HIV-infected, 21% (n = 6) had hypertension, and 18% (n = 5) had diabetes mellitus. One patient was in her second trimester of pregnancy with no other significant history [39], one patient had a history of polycythemia vera and monoclonal gammopathy of unknown significance [24], whereas two patients reported close contact with pigeons [41,46]. Of those with HIV, absolute CD4 count ranged from 0 to 157 cells/μL [9,28,36,42,43,50], whereas only three patients were noted to be on ART [36,42]. Six patients, all of whom were HIV-infected, were previously treated for CM which occurred from 2 to 22 months prior to their diagnosis of cerebral cryptococcoma [9,36,42,53]. 

### 3.2. Causative Pathogens

*C*. *neoformans* was responsible for 74% of the 34 cases in which an organism was isolated and speciated [19,22,23,24,26,27,28,29,30,32,34,36,37,39,41,44,45,46,47,50,52,53,54]. Among the nine cases which implicated *C*. *gattii* as the infectious etiology, only four provided explicit details as to the method for identifying the organism, either via isolation from a clinical specimen or genotypic testing [18,31,40,48]. Of the remaining 13 cases, *Cryptococcus* spp. was identified via histopathologic examination following biopsies or surgical resection of the cerebral lesions [20,25,33,38,42,55,56], except for 4 cases from a single case series published in 1990 where *Cryptococcus* spp. was isolated in culture but not speciated [43]. 

### 3.3. Clinical Manifestations

Patients with cerebral cryptococcomas most often presented with headache (58%, n = 26), altered mental status and/or confusion (38%, n = 17), and vomiting (31%, n = 14). Less common manifestations included fever (18%, n = 8), drowsiness or fatigue (16%, n = 7), seizures (13%, n = 6), and visual disturbances or blurry vision (11%, n = 5). On physical examination, 18% (n = 8) were noted to have papilledema, whereas 16% (n = 7) and 11% (n = 5) were observed to have upper and lower extremity weakness, respectively. Time from symptom onset to presentation ranged from 1 day to 365 days (median 30 days).

### 3.4. Site and Description of Lesion(s)

In 66% (n = 31) of cases, magnetic resonance imaging (MRI) was used to identify cerebral cryptococcomas, of which 74% (n = 24) utilized gadolinium. A single cerebral cryptococcoma lesion was present in 53% (n = 25) of patients, whereas multiple lesions were identified in 28% (n = 13) of cases. Cryptococcomas were most often detected in the frontal lobe (21%, n = 9), basal ganglia (21%, n = 9), and parietal lobe (19%, n = 8). One patient also had evidence of a concomitant T11-12 cryptococcoma [18]. Perilesional edema was present in 73% (n = 30) of patients, whereas hydrocephalus was identified in 38% (n = 9).

### 3.5. Treatment Details

Treatment details were provided for 96% (n = 45) of patients, as two patients died prior to surgical or pharmacological intervention [36,39]. Forty-nine percent (n = 22) of patients underwent surgical resection of one or more lesions, in which one patient underwent complete surgical resection of the lesion and antifungal therapy was not administered post-operatively [20]. Notably, surgical intervention occurred more often in those with lesions measuring 3 or more centimeters. Alternatively, surgical intervention was performed less often in patients with more than one lesion (38%) despite high rates of perilesional edema (71%). Management of intracranial pressure (ICP) was described in only a few cases, but of those, ventricular shunts were placed in three patients [18,22,43], whereas another two patients underwent external ventricular drain (EVD) implantation [42,52] as a result of failure to control ICP with serial lumbar punctures (LPs) [26], clinical deterioration [30], or development of hydrocephalus [50].

Of the 44 patients who received antifungal therapy, “induction” regimens most often consisted of an amphotericin B-based formulation (91%, n = 40) combined with flucytosine or fluconazole in 45% (n = 18) or 13% (n = 5), respectively. The duration of “induction” antifungal therapy varied widely from 7 to 180 days (median 42 days). The specific amphotericin B formulation utilized was included in 70% (n = 28) of cases, whereas specific doses, including mg/kg/day or total mg/dose, were reported in 53% (n = 21). Among patients who received an amphotericin B-based formulation, the median duration of therapy was 38 days (range 7–180 days), but details were limited as to why some treatment durations were abbreviated or prolonged. However, in one patient, amphotericin B was discontinued after the development of hyperkalemia, hypomagnesemia, and a pruritic rash, all of which improved after the initiation of fluconazole [33]. Fluconazole was exclusively administered to patients who did not receive an amphotericin B-based formulation as “induction” therapy for a median duration of 56 days (range 32 to 84 days).

Antifungal therapy was continued as “maintenance” therapy in 52% (n = 23) of patients who received “induction” therapy. Fluconazole was most often administered (83%, n = 19), followed by voriconazole (17%, n = 4). Fluconazole doses ranged from 200 to 1200 mg/day [25,27,28], whereas voriconazole 200 to 300 mg twice daily was most often used [18]. One patient received, every other day, amphotericin B deoxycholate (AmB-d) as “maintenance” therapy [19]. The duration of “maintenance” antifungal therapy ranged from 60 to 510 days (median 126 days), with one patient receiving lifelong fluconazole [50]. Two patients received localized therapy whereby one patient received intrathecal amphotericin B [43], whereas AmB-d was directly administered into the abscess cavity in the other [21]. 

Corticosteroids were administered to 27% (n = 12) of the 44 patients who received “induction” antifungal therapy for a median of 31.5 days (range 1–60 days). The specific corticosteroid and dose utilized was often not mentioned, but where described, dexamethasone 12 to 28 mg/day in three to four divided doses were most frequently administered [18,42,50]. The rationale for starting corticosteroids were provided in very few cases, and included initiation during the early phases of antifungal therapy, as well as later on in the treatment course in response to complications, such as increased ICP despite serial LPs [42], presence of hydrocephalus on repeat imaging [42], spinal cord edema, and paradoxical immune reconstitution inflammatory syndrome (IRIS) during week 8 of antifungal therapy [18]. Adjunctive medications, besides corticosteroids, were not widely used. One patient was treated with interferon (INF)-γ three times per week after 3 weeks of antifungal therapy for refractory disease [18], whereas another was started on subcutaneous adalimumab every 2 weeks due to clinical deterioration despite antifungal therapy [9].

### 3.6. Outcomes

Mortality was 31% (n = 14) among the 45 patients who underwent surgical or pharmacological intervention. Causes of death included refractory hydrocephalus [22], *Pneumocystis jirovecii* pneumonia [43], sepsis or septic shock [32,55], post-operative cardiovascular complications [45], as well as torsades de pointes secondary to fluconazole [35]. Time to death ranged from 2 to 420 days (median 25.5 days). 

Patients who died were more likely to report a significant past medical or surgical history (64% vs. 40%), be infected with *C*. *neoformans* (69% vs. 45%), present with altered mental status or confusion (53% vs. 30%), nuchal rigidity (20% vs. 0%), as well as upper or lower extremity weakness (27% and 27% vs. 3% and 10%, respectively) compared to survivors. The presence of multiple lesions and perilesional edema were similar between groups. However, fewer patients that died underwent surgery (40% vs. 53%). Though an amphotericin B-based formulation was commonly administered as “induction” therapy in both groups (80% vs. 90%), those who died received a shorter duration of “induction” therapy (median, 21 days (range, 10–42 days) vs. 42 days (range, 7–180 days)). In addition, the duration of “maintenance” therapy was much shorter in those who died (median, 60 days (range, 6–76 days) vs. 180 days (range, 60–730 days)), despite 90% of patients in each group receiving fluconazole or voriconazole. Approximately 40% of patients in each group received corticosteroids, but the median duration of therapy was 4.5 days (range, 1–8 days) in patients who died compared to 49 days (range, 21 to 60 days) in those who survived. 

Of the 31 patients who survived, 94% (n = 29) were described as having symptomatic, clinical, and/or radiographic improvement during follow-up. Median time to follow-up was 255 days (range, 7 to 4380 days). Though most patients were asymptomatic at follow-up, one described residual neuromotor symptoms on day 1460 [25], whereas another patient experienced improvement in symptoms initially, but later required readmission due to worsening neurologic symptoms [48]. 

Few cases described recurrent disease during the follow-up period [22,28,29,30,41,42,48], with even fewer detailing subsequent surgical or pharmacological intervention. An HIV-infected patient was asymptomatic with near complete radiographic resolution after 7 months of antifungal therapy and 5 months of ART, but then experienced a recurrence with worsening basal ganglia lesions on MRI, prompting initiation of combination antifungal therapy and corticosteroids for 6 weeks, followed by long-term fluconazole [28]. Radiographic improvement was noted after 6 months of treatment, but the patient experienced another recurrence 4 months later despite remaining on fluconazole and ART. The patient was treated with combination antifungal therapy for 6 weeks, then transitioned to long-term voriconazole, instead of fluconazole, without the development of new symptoms after 10 months. In another case, a patient who underwent surgical resection of left frontal lobe and left temporal lobe cryptococcomas followed by antifungal therapy, presented after 6 months due to non-adherence with new onset headaches and upper extremity weakness with the identification of two right parietal lobe lesions [29,30]. The patient underwent surgical resection, and was then started on oral fluconazole with reported symptom improvement after 8 weeks. 

## 4. Discussion

In this systematic scoping review, we present a thorough analysis of the data, describing the demographic characteristics, diagnostic findings, treatment modalities, as well as outcomes for 47 individual patients with cerebral cryptococcomas. Though data presented across all 40 records were quite heterogeneous, as all but three were case reports, this review provides valuable insight into an understudied, and perhaps under recognized, area with limited guideline recommendations due to low quality evidence [13,14,15] despite an increasing frequency of at-risk patients [2,3,4,5,6,7,8]. As a result, findings from this systematic scoping review may lead to improvements in patient care and better clinical outcomes.

Though previous data suggest cerebral cryptococcomas are most often caused by *C*. *gattii* [14], *C*. *neoformans* was isolated more often than *C*. *gattii* in our analysis (Table 2). However, in 27% (n = 13) of cases, the cryptococcal isolate was not speciated, largely due to identification via histopathologic examination, which cannot distinguish between *C*. *neoformans* and *C*. *gattii*. Furthermore, *C*. *gattii* was initially recognized as a variant of *C*. *neoformans*, and later recognized as an independent species [57]. *C*. *gattii* was recently reclassified as a species complex composed of four individual species. Improved methods to differentiate *C*. *gattii* from *C*. *neoformans* over the recent years have led to greater recognition of the global environmental distribution of *C*. *gattii*. Notably, all the cases where *C*. *gattii* was detected were published between 2005 and 2017 [18,21,31,35,40,48,49,51,52]. Due to resource limitations to perform species identification in many laboratories, it is likely that *C*. *gattii* may be responsible for some of the cases attributed to *C*. *neoformans* presented in this review.

Cerebral cryptococcomas are challenging to diagnose, and are often missed on initial evaluation due to their highly variable clinical presentation in patients with and without comorbidities or risk factor for cryptococcosis. Similar to epidemiologic findings in patients with CM [58], males comprised 75% of the cases included in our analyses. Almost 80% were at least 30 years of age, in which 14% of patients were aged 30 to 39 years, 29% were 40 to 49, 29% were 50 to 59, 14% were 60 to 69, and 14% were over 70 years. Overall, most patients reported a past medical or surgical history including known risk factors for cryptococcosis, such as HIV-infection, diabetes, or idiopathic CD4 lymphocytopenia [59]. Patients with *C*. *gattii* were less likely to report a significant past medical or surgical history, whereas those with *C*. *neoformans* were more likely to be HIV-infected, which is similar to findings from previous data [58,60]. A few patients reported social histories with established ecologic risk factors, including living in or traveling to Australia in the case of *C*. *gattii* [18,49], and exposure to birds and bird droppings in the case of *C*. *neoformans* [41,46]. Patients with cerebral cryptococcomas presented with a wide range of often non-specific symptoms and physical exam findings, from headache and altered mental status to vomiting, giddiness, word-finding difficulties, and cerebellar signs, which were more common among patients 50 years and older. Clinical manifestations differed between patients with *C*. *neoformans* and those with *C*. *gattii* (Table 2).

**Figure 1:**
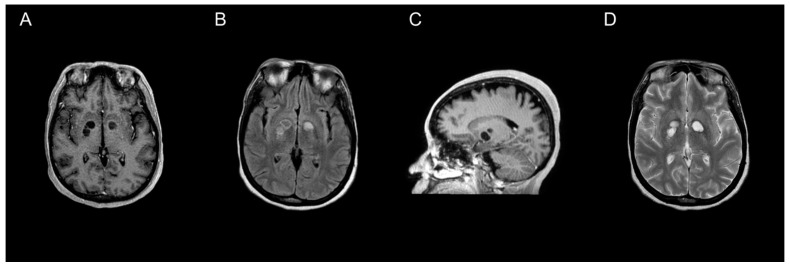
MRI characterization of cryptococcomas. MRI of the brain showing a nonenhancing cryptococcoma (axial plane T1-weighted post contrast (**A**), axial plane T2-FLAIR (**B**), post-contrast parasagittal (**C**), and axial plane T2-weighted (**D**)).

All 47 individual patients included in our review underwent neuroimaging, of which MRI was utilized more often than CT (66% vs. 34%, respectively). Though CTs are often preferentially performed due to availability and timeliness [61], previous data suggest increased sensitivity for detecting cryptococcoma lesions using gadolinium-enhanced MRI compared to standard MRI or CT [10,54,61]. MRI findings vary, ranging from hyper-intense on T2-weighted images, to non-enhancing on postcontrast T1-weighted images (Figure 1). Due to the rarity of cerebral cryptococcomas and challenges associated with diagnosis, it is common for them to be mistaken for neoplasms, as well as pyogenic or tubercular abscesses, prior to surgery [52]. Less often, cerebral cryptococcomas have been misdiagnosed as neurocysticercosis [39], or even a tumefactive demyelinating lesion, a rare focal demyelinating disease [62]. Cryptococcomas are sometimes confused in neuroimaging with dilated perivascular spaces (Virchow–Robin spaces) that coalesce to form gelatinous pseudocysts [63]. However, cryptococcomas resulting from invasion of *Cryptococcus* spp. into the brain parenchyma may develop in a variety of locations throughout the brain [10,11,52]. The frontal and parietal lobes (21% and 19%, respectively), as well as the basal ganglia (21%) were most often involved, whereas the thalamus (5%) or the pons (2%) were rarely involved among the 47 individual patients included in our analysis. The median number of lesions identified was one, but ranged from one to three among reports that provided specified details. Forty-seven percent of patients were noted to have more than one or multiple lesions throughout the brain parenchyma, which was more common amongst patients with *C*. *gattii* (Table 2). Characteristics of the lesions were not universally reported, but the size of the cryptococcomas varied substantially from less than 1 cm to 5 or 6 cm. *C*. *neoformans* was identified more often among cases where measurements were provided [23,24,34,44,50]. Perilesional edema and hydrocephalus were slightly more common among patients with *C*. *neoformans* than those with *C*. *gattii* (Table 2). Intracranial imaging is a valuable tool to determine the extent of disease severity in patients with disseminated cryptococcosis, but is insufficient to establish a diagnosis of cerebral cryptococcoma. 

Amphotericin B and flucytosine followed by long-term fluconazole remains the mainstay of treatment in CM [13,14,15]. Treatment strategies for patients with CM have largely been extrapolated to patients with cerebral cryptococcomas, as no prospective studies have been performed. Though guidelines suggest initial therapy for patients with cerebral cryptococcoma should include lipid-associated formulation of amphotericin B, in lieu of AmB-d when available, in combination with oral flucytosine for at least 42 days followed by oral fluconazole 400 mg to 800 mg per day for 180 to 540 days [14,15], regimen selection and duration were inconsistent across all most cases. More than 90% of patients received an amphotericin B-based formulation, of which 58% received combination therapy, whereas only 52% received “maintenance” therapy. Details about the specific formulation and/or dose utilized and associated rationale for “induction” and “maintenance” antifungal therapies were rarely provided. 

Approximately 25% of all patients received corticosteroids, of which only 50% had perilesional edema. Though limited details regarding the specific corticosteroid and/or dose administered were reported, the median duration of corticosteroid therapy was 21 days (range, 1–60 days) among patients with perilesional edema. In addition, details regarding corticosteroid tapers were also not provided. Although guidelines mention corticosteroids in the setting of cerebral cryptococcomas with surrounding edema, no recommendations for which specific corticosteroid and/or dose are available [14,15]. 

Of the 47 individual patients included in this analysis, 34% died prior to undergoing surgical or pharmacological intervention, discharge, or during the follow-up period, similar to that observed with CM [1]. Fewer patients who underwent surgical intervention died, which may necessitate greater emphasis on the role of surgery as a component of the overall management for cerebral cryptococcomas in the guidelines [13,14,15]. In addition, prolonged durations of “induction” and “maintenance” therapy were reported more often in patients who survived, and should continue to be considered the standard of care. However, the guidelines recommend a “gradual reduction” in corticosteroids [14,15], but the vague recommendations should be modified to emphasize the importance that the tapering should occur over at least a 3-to-6-week period.

## 5. Limitations

Limitations of this systematic scoping review must be acknowledged. First, we chose to complete a scoping systematic review due to the lack of an available comprehensive review about cerebral cryptococcomas. Second, our analysis only included 47 individual patients derived from 40 reports. In order to abstract consistent data each report, we had to exclude publications that did not provide information on the site of infection; treatment regimen at least including the specific antifungal therapy administered, if applicable; and patient outcome. Third, specific details about the pharmacological treatments administered, such as the route, dose, and/or duration, were often not reported, which limited our abilities to provide clear treatment recommendations, but may be due to the paucity of recommendations provided in the guidelines [13,14,15]. Additionally, few reports clearly delineated “induction” therapy versus “maintenance” therapy. Fourth, the duration of “induction”, “maintenance”, and corticosteroid therapy was longer in patients who survived than those who died, which could represent survival-related selection bias. Lastly, due to the inherent difficulties of diagnosing cerebral cryptococcomas, many additional cases may be unrecognized and, therefore, not reported. 

## 6. Conclusions

Despite guideline recommendations, diagnosis and treatment of cerebral cryptococcomas is very heterogeneous due to the lack of high-quality data. In our analysis, *C*. *neoformans* was identified more often than *C*. *gattii* as the causative pathogen in cases where an organism was isolated and speciated, which may be due to difficulties differentiating *C*. *neoformans* from *C*. *gattii*, and underreporting. Furthermore, cerebral cryptococcomas are most often identified as a single lesion with surrounding edema on gadolinium-enhanced MRI of the brain, and occur more often in male patients over 30 years of age who frequently report a significant past medical, surgical, or social history, and present with non-specific symptoms and physical exam findings. Though the most efficacious treatment remains undefined, a multipronged approach should be utilized that includes corticosteroids in the setting of perilesional edema tapered over 3 to 6 weeks; considerations for surgical intervention, if feasible; as well as a prolonged duration of antifungal therapy. Emphasis should be placed on providing species identification and well-documented treatment details in future reports to allow for the development of more concise evidence-based recommendations.

## Figures and Tables

**Table 1:** Reported cases of cerebral cryptococcomas.

Case	Location	Age (Years)	Sex	Medical, Surgical, or Social History	Causative Pathogen	Clinical Manifestations, Duration	Number and Location of Lesion(s)	Treatment Course	Outcome
Amburgy et al., 2016 [18]	U.S.	Middle age ^†^	M	Cocaine use, travel to Hawaii, Philippines, Thailand, Australia, Japan, and China over the last 30 years, otherwise unremarkable	*C*. *gattii*	Fevers, chills, headache, back pain, vomiting, 28 days	Multiple: basal ganglia, and subcortical white matter (also had evidence of a T11-12 cryptococcoma)	ABLC 5–6 mg/kg/day + flucytosine x 83 days Serial LPs then VPS placed INF-γ three times per week started during week 3 for refractory disease Dexamethasone 4 mg every 6 h × 10 days during week 8 for spinal cord edema and paradoxical IRIS Oral voriconazole 300 mg twice daily and dexamethasone taper as maintenance therapy	Improving white matter lesions and no evidence of spinal lesion on MRI after 10 days of dexamethasone
Bayardelle, et al., 1982 [19]	Canada	42	M	Unremarkable	*C*. *neoformans*	Headache, seizure, 30 days	3: upper L parietal region, R rolandic area, cerebral parenchyma posterior to the frontal opercular region	AmB-d 20 mg/day + flucytosine 150 mg/kg/day × 6 weeks, then AmB-d 50 mg every other day monotherapy until total dose of 2160 mg	Asymptomatic with near complete regression of cerebral lesions on CT at 1-year follow-up
Brunasso et al., 2021 [20]	Italy	32	F	Tonic-clonic seizures	*Cryptococcus* spp.	Asymptomatic	1: R temporo-mesial lesion	Complete surgical resection without antifungal therapy	Asymptomatic at 6-month follow-up
Colom et al., 2005 [21]	Spain	60	M	Diabetes mellitus	*C*. *gattii*	Headaches, somnolence, several days	1: basal ganglia	L-AMB 200 mg/day + fluconazole 800 mg/day + flucytosine 2500 mg every 6 h × 6 months AmB-d was administered directly into the abscess at 3 months Oral voriconazole as maintenance therapy	Asymptomatic at 16-month follow-up
Coppens, et al., 2006 [22]	Belgium	63	M	Diabetes mellitus	*C*. *neoformans*	Weight loss, fatigue, headache, somnolence, hemianopsia, disorientation to time and place, 210 days	3: R parietal, R frontal, and L occipital lobes	AmB-d 0.7 mg/kg/day + flucytosine 150 mg/kg/day × 2 weeks, then transitioned to oral fluconazole 400 mg/day × 3 weeks Underwent intraventricular drain placement into L occipital abscess due to mental deterioration, then replaced by VPS and transitioned to IV voriconazole 400 mg every 12 h × 1 day then 200 mg every 12 h thereafter	MRI at 3-month follow-up showed reduction of initial mass, dural enhancement and thickening, new contrast enhancing lesions in the brain parenchyma, and cortical necrosis Died due to refractory hydrocephalus uncontrolled cryptococcosis
Guha, et al., 2015 [23]	U.S.	66	F	Hypertension, diabetes mellitus, hyperlipidemia	*C*. *neoformans*	Global limb weakness, anorexia, cough, seizures, night sweats, 7 days	1: postcentral gyrus (1.1 cm)	Surgical resection of the lesion followed by L-AMB × 6 weeks, then fluconazole × 1 year	Improved neurologic function and no new symptoms at 1-year follow-up
Guhjjar et al., 2021 [24]	U.S.	58	M	JAK2 positive polycythemia vera, MGUS, hypertension	*C*. *neoformans*	Confusion, drowsiness, auditory hallucinations, L sided weakness, 7 days	1: R basal ganglia (0.8 × 0.5 cm)	L-AMB + flucytosine × 6 days, then discharged to receive fluconazole	Remained on fluconazole with improved neurologic function and no new symptoms at 2-year follow-up
Hagan et al., 2014 [25]	Brazil	25	F	Unremarkable	*Cryptococcus* spp.	Word-finding difficulty, R sided numbness and weakness	1: L thalamus (3 × 2 cm)	Amphotericin B, followed by IV fluconazole 800 mg/day × 2 months, then 600 mg/day × 8 months, then 300 mg/day × 9 months	Complete resolution of lesion on MRI, mild residual neuromotor symptoms at 4-year follow-up
Hiraga et al., 2015 [26]	Japan	71	F	Hypertension, hyperthyroidism	*C*. *neoformans*	R lower limb weakness, headache, loss of appetite, diplopia, 3 days	1: L frontal lobe	L-AMB + flucytosine	Died 20 days after hospitalization
Ho et al., 2005 [27]	Taiwan	55	F	Unremarkable	*C*. *neoformans*	Headache, facial palsy, 365 days	1: R frontal lobe	Surgical resection of the lesion, followed by AmB-d 0.6–0.7 mg/kg/day + fluconazole 400 mg/day × 10 weeks	No new symptoms at 8-month follow-up
Hu et al., 2013 [28]	China	19	M	HIV-infected (CD4 0 cells/μL)	*C*. *neoformans*	Headache, confusion, 14 days	Bilateral basal ganglia	AmB-d 0.7 mg/kg/day + flucytosine 100 mg/kg/day × 11 weeks, followed by fluconazole 400 mg/day as maintenance therapy	Asymptomatic with near complete resolution of lesions on MRI after 6 months of antifungal therapy and 4 months of ART 1 month later, remained on fluconazole 400 mg/day and ART, MRI with worsening lesions in bilateral basal ganglia treated with AmB-d + voriconazole 200 mg every 12 h + flucytosine × 6 weeks, followed by fluconazole 400 mg/day combined with corticosteroids over a 6-month period resulting in near complete resolution of brain lesions 15 months after ART initiation while on fluconazole, MRI demonstrated new L temporal lobe lesions treated with AmB-d + voriconazole + flucytosine × 6 weeks, followed by voriconazole 200 mg every 12 h No new symptoms at 10-month follow-up while remaining on voriconazole
Kelly et al., 2018 [29] and Kelly et al., 2020 [30]	South Africa	19	M	Unremarkable	*C*. *neoformans*	Headache, blurry vision, R upper extremity weakness, tonic-clonic seizure	2: L frontal lobe, temporal lobe	Surgical resection of the lesions, followed by oral fluconazole 800 mg/day × 8 weeks	Non-adherent to antifungal therapy × 6 months post-discharge, then represented with headache and L upper extremity weakness due to 2 R parietal lobe lesions for which he underwent surgical resection, followed by oral fluconazole 800 mg/day × 8 weeks with symptom improvement [30]
King et al., 2014 [31]	Australia	59	M	Unremarkable	*C*. *gattii*	Flashing lights and intermittent blindness in R eye, 270 days	2: R temporal lobe, L occipital lobe	Surgical resection of both lesions, followed by amphotericin B + flucytosine × 4 weeks, then oral antifungal therapy ^†^ × 12 months	Resolution of lesions on MRI at 12-month follow-up
Krishnan et al., 2004 [32]	Australia	72	M	Parkinson’s disease, diabetes mellitus	*C*. *neoformans*	Depression, confusion, falls, 42 days	2: L parietal lobe, R superior cerebellar peduncle	Amphotericin B × 4 weeks, followed by oral fluconazole as maintenance therapy	Mild improvement in mental state initially, but died 2 months later due to septic shock
Kumar et al., 2020 [33]	India	48	M	Unremarkable	*Cryptococcus* spp.	Headache, giddiness, vomiting, bilateral papilledema, 90 days	1: cerebellar hemisphere (3 × 3 × 4 cm)	Surgical resection of the lesion, followed by AmB-d 1 mg/kg/day, then fluconazole 400 mg/day × 10 weeks total	Clinical improvement and resolution of lesion on MRI at 5-month follow-up
Li et al., 2010 [34]	China	49	F	Unremarkable	*C*. *neoformans*	Headache, dizziness, vomiting, 30 days	1: R occipital lobe (5 × 4 × 4.5 cm)	Surgical resection of the lesion, followed by AmB-d 25 mg/day × 20 days	Resolution of symptoms and lesion on MRI at 1-month follow-up
McMahon et al., 2008 [35]	Australia	68	F	Hypertension	*C*. *gattii*	Falls, 30 days	2: L pons and middle cerebellar peduncle	ABLC + flucytosine × 6 weeks, then fluconazole 400 mg/day to 600 mg/day	Died 48 days after treatment initiation from torsades de pointes attributed to fluconazole
Musubire et al., 2012 [36]	Uganda	35	M	HIV-infected on ART (VL UD, CD4 89 cells/μL), treated for CM 7 months prior	*C*. *neoformans*	Headache, photophobia, dizziness, anorexia, behavioral changes	1: R parietal lobe	Died prior to surgery and antifungal therapy initiation	Not applicable
Nadkarni et al., 2005 [37]	India	22	M	Seizures	*C*. *neoformans*	Seizures, L hemiparesis, bilateral papilledema	1: R parietal lobe	Surgical resection of the lesion, followed by L-AMB	Resolution of symptoms at 9-month follow-up
Nakwan et al., 2009 [38]	Thailand	23	M	Migraine headaches	*Cryptococcus* spp.	Headache, vomiting, ataxia, dysmetria, dysdiadochokinesia, 365 days	Multiple: cerebellum	Surgical resection of the lesion, followed by amphotericin B × 4 weeks, then oral fluconazole 800 mg/day × 6 months	Resolution of symptoms at 6-month follow-up
Nucci et al., 1999 [39]	Brazil	29	F	Pregnant (2^nd^ trimester)	*C*. *neoformans*	Sleepiness, vomiting, bilateral 6^th^ nerve palsy, nuchal rigidity, papilledema, 120 days	Multiple: basal ganglia, R lateral ventricle	Diagnosis established postmortem 9 days after presentation	Not applicable
Oliveira et al., 2007 [40]	Brazil	64	M	Unremarkable	*C*. *gattii*	Fever, anorexia, disorientation, weakness, bilateral papilledema, 7 days	1: R temporal lobe, multiple nodules throughout brain parenchyma	Aspiration of temporal lobe lesion, followed by amphotericin B + dexamethasone	Died 2 days after treatment initiation
Paiva et al., 2017 [41]	Brazil	54	F	Hypertension, direct contact with several bird species including pigeons	*C*. *neoformans*	Behavioral disturbances, confusion, weakness, 60 days	2: L occipital lobe	Surgical resection of the lesions, followed by amphotericin B + fluconazole	Died from disease and medication related complications
Pettersen et al., 2015 [42]	U.S.	30	M	HIV-infected on ART (CD4 157 cells/µL), treated for recurrent CM 2 months prior	*Cryptococcus* spp.	Headache, fever, nuchal rigidity, night sweats, seizures	Multiple: R caudate, R temporal lobe	L-AMB 3 mg/kg/day + flucytosine 25 mg/kg every 6 h × 2 weeks EVD placed and prednisone 60 mg/day started due to hydrocephalus on repeat CT Fluconazole 1200 mg/day and prednisone taper as maintenance therapy	Calcification of the caudate head without evidence of cryptococcoma on CT at 2-week follow-up, but eventually transitioned to hospice
Pettersen et al., 2015 [42]	U.S.	40	M	HIV-infected on ART (CD4 84 cells/µL), treated for CM 3 months prior	*Cryptococcus* spp.	Headache, expressive aphasia, R facial weakness, weight loss	2: L frontotemporal region	L-AMB 5 mg/kg/day + flucytosine 21 mg/kg every 6 h × 6 weeks Oral dexamethasone 4 mg every 8 h started due to increased ICP despite serial LPs Fluconazole 800 mg/day as maintenance therapy for up to 18 months	Resolution of symptoms and lesions on MRI at 2-month follow-up
Popovich et al., 1990 [43]	U.S.	52	M	HIV-infected	*Cryptococcus* spp.	Headache, altered mental status, photophobia, nausea, vomiting, 1 day	Multiple: bilateral cerebral hemispheres	Amphotericin B	Resolution of symptoms during hospitalization and gradual resolution of lesions on CT
Popovich et al., 1990 [43]	U.S.	47	F	Unremarkable	*Cryptococcus* spp.	Headache, nausea, vomiting, somnolence, L hemianopsia, 3 days	1: temporal horn of R lateral ventricle	Craniotomy and R temporal lobe incision with placement of ventriculojugular shunt, followed by amphotericin B + flucytosine	Resolution of symptoms at discharge
Popovich et al., 1990 [43]	U.S.	30	M	HIV-infected, previously treated for CM	*Cryptococcus* spp.	Headache, nausea, vomiting, 28 days	Multiple: bilateral basal ganglia	Amphotericin B + flucytosine	Decrease in size and number of lesions on CT several months after discharge
Popovich et al., 1990 [43]	U.S.	50	M	HIV-infected, treated for CM 2 months prior	*Cryptococcus* spp.	Not specified	Multiple: bilateral thalamus and basal ganglia	Amphotericin B + intrathecal amphotericin B	Resolution of symptoms at discharge, but died 1 month later due to PJP
Rai et al., 2012 [44]	India	50	M	Idiopathic CD4 lymphocytopenia (CD4 204 cells/µL)	*C*. *neoformans*	Headache, dysmetria, dysdiadochokinesia, impaired gait, impaired gag reflex, weak hand grip, 365 days	2: vermis (largest 3.25 × 3.18 × 3.16 cm)	Craniotomy and aspiration of the larger lesion, followed by L-AMB + corticosteroid taper × 8 weeks with addition of fluconazole after 4 weeks, followed by fluconazole + flucytosine × additional 2 weeks, then oral fluconazole as maintenance therapy	Reduction in lesion size and symptom improvement after 10 weeks of treatment
Sabbatani, et al., 2004 [45]	Italy	46	M	Homocystinuria, renal dysfunction, anemia	*C*. *neoformans*	Time–space disorientation	1: R frontal lobe	Surgical resection, followed by IV fluconazole 600 mg/day × 32 days, then IV voriconazole 400 mg/day × 60 days	Residual cerebral damage on MRI and progressive cognitive decline at 14-month follow-up, but died due to post-operative cardiovascular complications
Saigal et al., 2005 [46]	U.S.	49	M	Cleaned pigeon droppings from coop 1 month prior to presentation, otherwise unremarkable	*C*. *neoformans*	Headache, syncope, confusion, mental status changes, 30 days	Multiple: bilateral basal ganglia	Amphotericin B × 2 months, followed by fluconazole + flucytosine + corticosteroids × 2 months	Clinical improvement and resolution of lesions on MRI at 2-year follow-up
Santander et al., 2019 [47]	Spain	41	F	Unremarkable	*C*. *neoformans*	Gait disturbance, urinary incontinence, impaired memory, 120 days	1: biventricular mass (1.6 cm diameter)	Surgical resection of the lesion, followed by amphotericin B 400 mg/day + IV flucytosine 1500 mg every 6 h	Died 10 days after antifungal therapy initiation
Sellers et al., 2012 [48]	U.S.	70	M	Unremarkable	*C*. *gattii*	Stupor, lethargy, 3 days	Multiple: bilateral basal ganglia	L-AMB + flucytosine x 4 weeks	Resolution of symptoms initially, but readmitted 1 week later with worsening neurologic symptoms
Sitapati et al., 2010 [9]	U.S.	28	M	HIV-infected (CD4 149 cells/μL), treated for CM 22 months prior	*Cryptococcus* spp.	Seizures, expressive aphasia, R sided weakness	1: L temporal lobe (6.0 × 3.4 × 3.3 cm)	L-AMB + flucytosine + IV dexamethasone × 1 week, followed by 2-week dexamethasone taper + fluconazole 800 mg/day as maintenance therapy Clinically deteriorated over the next 5 months prompting administration of adalimumab 40 mg SQ every 2 weeks	No change in lesion on MRI after 4 weeks of adalimumab Cognitive and motor improvement after 10 weeks of adalimumab
Solis et al., 2017 [49]	Australia	54	M	Worked with timber in New South Wales, Australia, otherwise unremarkable	*C*. *gattii*	Dysarthria, L facial droop	1: R frontal lobe (1.9 × 3.0 × 2.5 cm)	Surgical resection, followed by L-AMB 3 mg/kg/day × 4 weeks + flucytosine × 2 weeks, then transitioned to itraconazole × 12 months as maintenance therapy	Resolution of lesion on MRI and symptoms at 4-month and 9-month follow-up, respectively
Troncoso, et al., 2002 [50]	Argentina	28	M	HIV-infected (CD4 28 cells/μL)	*C*. *neoformans*	Fever, headache, hallucinations, altered mental status, seizures, 14 days	1: L occipital lobe (2 cm)	IV AmB-d 0.7 mg/kg/day + dexamethasone 28 mg/day × 6 weeks, followed by lifelong oral fluconazole 200 mg/day	Discharged after 8 weeks following subjective and objective improvement; MRI at 1-year follow-up demonstrated reduction in size of lesion
Ulett et al., 2017 [51]	Australia	55	M	Hypertension, gout, diabetes mellitus	*C*. *gattii*	Headache, R papilledema, L pronator drift, 30 days	1: R frontoparietal (4 × 5 × 4.8 cm)	Surgical resection, followed by ABLC 5 mg/kg/day + flucytosine 66 mg/kg/day + dexamethasone taper × 34 days, then oral fluconazole 800 mg/day × 9 months	Improvement in lesion on MRI at 10-month follow-up
Uppar, et al., 2018 [52]	India	55	M	Unremarkable	*C*. *neoformans*	Fever, altered sensorium, headache, vomiting, behavioral changes, hemiparesis, papilledema, L 6th nerve palsy, L upper motor neuron facial palsy	1: R parieto-occipital lobe	Surgical resection, followed byAmB-d 1 mg/kg/day × 6 weeks + oral fluconazole 400 mg/day × 18 weeks	Healthy at 8-year follow-up
Uppar, et al., 2018 [52]	India	45	M	Unremarkable	*C*. *neoformans*	Giddiness, headache, vomiting, cerebellar signs	1: R cerebellum	Surgical resection with EVD placement, followed by AmB-d 1 mg/kg/day × 8 weeks + oral fluconazole 400 mg/day × 18 weeks	Healthy at 12-year follow-up
Uppar, et al., 2018 [52]	India	74	M	Unremarkable	*C*. *gattii*	Headache, reduced appetite, vomiting, cerebellar signs	1: R cerebellum	Surgical resection, followed by AmB-d 1 mg/kg/day × 6 weeks + oral fluconazole 400 mg/day × 18 weeks	Healthy at 4-year follow-up
Uppar, et al., 2018 [52]	India	30	M	Unremarkable	*C*. *neoformans*	Headache, vomiting, fever, visual disturbances, papilledema	1: R frontal lobe	AmB-d 1 mg/kg/day × 6 weeks + oral fluconazole 400 mg/day × 18 weeks	Healthy at 6-month follow-up
Uppar, et al., 2018 [52]	India	24	M	Unremarkable	*C*. *neoformans*	Headache, vomiting, fever, behavioral changes, altered sensorium, visual disturbances, papilledema, bilateral 6th nerve palsy	1: R caudate region	Surgical resection, followed by AmB-d 1 mg/kg/day × 6 weeks + oral fluconazole 400 mg/day × 8 weeks	Died 2 months following surgery
Velamakanni et al., 2014 [53]	Uganda	45	M	HIV-infected (CD4 4 cells/µL), treated for CM 2 months prior	*C*. *neoformans*	Headache, cough, vomiting, fever, seizures, R-sided hemiparesis, 7 days	1: occipital lobe	AmB-d 50 mg/day, in addition to prednisone started on day 11	Died 2 weeks after diagnosis of cryptococcoma
Wei, et al., 2020 [54]	China	40	M	Unremarkable	*C*. *neoformans*	Altered consciousness, apathy, 7 days	Multiple: corpus callosum, centrum ovale	Initially received IV methylprednisolone 500 mg/day × 3 weeks, followed by prednisone 60 mg/day tapered over by 5 mg/week Clinically deteriorated after 3 months, then treated with AmB-d 25 mg/day increased to 50 mg/day + oral flucytosine 6 g/day	Died 3 weeks following antifungal therapy initiation
Yeh, et al., 2014 [55]	Taiwan	75	M	Unremarkable	*Cryptococcus* spp.	R sided weakness, several days	1: L parietal lobe	Surgical resection, followed by IV fluconazole	Died on post-operative day 17 from systemic sepsis
Zheng et al., 2011 [56]	China	53	F	Poultry farmer, otherwise unremarkable	*Cryptococcus* spp.	Headache, vomiting, ataxia, wide-based gait, dysmetria, 180 days	Multiple: posterior fossa	Surgical resection, followed by fluconazole x 12 weeks	Resolution of symptoms and decrease in size of lesions on MRI at follow-up

ABLC, amphotericin B lipid complex; AmB-d, amphotericin B deoxycholate; ART, antiretroviral therapy; CM, cryptococcal meningoencephalitis; EVD, external ventricular drain; HIV, human immunodeficiency virus; INF, interferon; IRIS, immune reconstitution inflammatory syndrome; IV, intravenous; JAK, Janus kinase 2; L, left; L-AMB, liposomal amphotericin B; MGUS, monoclonal gammopathy of undetermined significance; MRI, magnetic resonance imaging; PJP, *Pneumocystis jirovecii* pneumonia; R, right; UD, undetectable; U.S., United States; VPS, ventriculoperitoneal shunt; VL, HIV RNA viral load. ^†^, not specified.

**Table 2:** Comparisons between cerebral cryptococcomas caused by *C*. *neoformans* and *C*. *gattii*.

	*C*. *neoformans*	*C*. *gattii*
Prevalence *	74%	26%
Clinical manifestations		
Headache	56%	50%
Altered mental status or confusion	52%	25%
Visual disturbances	16%	13%
Seizures	16%	0%
Fever	20%	25%
Chills	0%	13%
Fatigue	16%	25%
Weight loss	4%	13%
Papilledema	20%	25%
Upper extremity weakness	20%	13%
Time from symptom onset to presentation (days), median (range)	30 (3–365)	29 (3–270)
Radiographic findings		
One or more lesions throughout the brain parenchyma	40%	56%
Perilesional edema	77%	47%
Hydrocephalus	71%	33%
Treatment regimens		
Amphotericin B-based formulation	88%	100%
Amphotericin B-based formulation in combination with flucytosine or fluconazole	57%	89%
“Induction” therapy duration among survivors (days), median (range)	42 (10–60)	38 (7–84)
“Maintenance” therapy duration among survivors (days), median (range)	126 (60–730)	317.5 (12–365)
Follow-up, median (range)	302.5 (30–4380)	279 (7–1460)

*, based on the 34 cases in which an organism was isolated and speciated [19,22,23,24,26,27,28,29,30,32,34,36,37,39,41,44,45,46,47,50,52,53,54].

## Data Availability

Not applicable.

## Supplementary Materials

The following supporting information can be downloaded at: https://www.mdpi.com/article/10.3390/pathogens11020205/s1. Figure S1: Preferred Reporting Items for Systematic Reviews and Meta-analyses (PRISMA) flow diagram.
