# Supplementary material for: Cerebral Cryptococcomas: A Systematic Scoping Review of Available Evidence to Facilitate Diagnosis and Treatment
Source: Pathogens. 2022 Feb 3;11(2):205. doi: 10.3390/pathogens11020205 (PMC8879191; doi:10.3390/pathogens11020205)
Supplement: Supplementary file 1 [file pathogens-11-00205-s001.zip › pathogens-1563736-supplementary.pdf]

Supplementary Figure S1. Preferred Reporting Items for Systematic Reviews and Meta-analyses (PRISMA) flow diagram

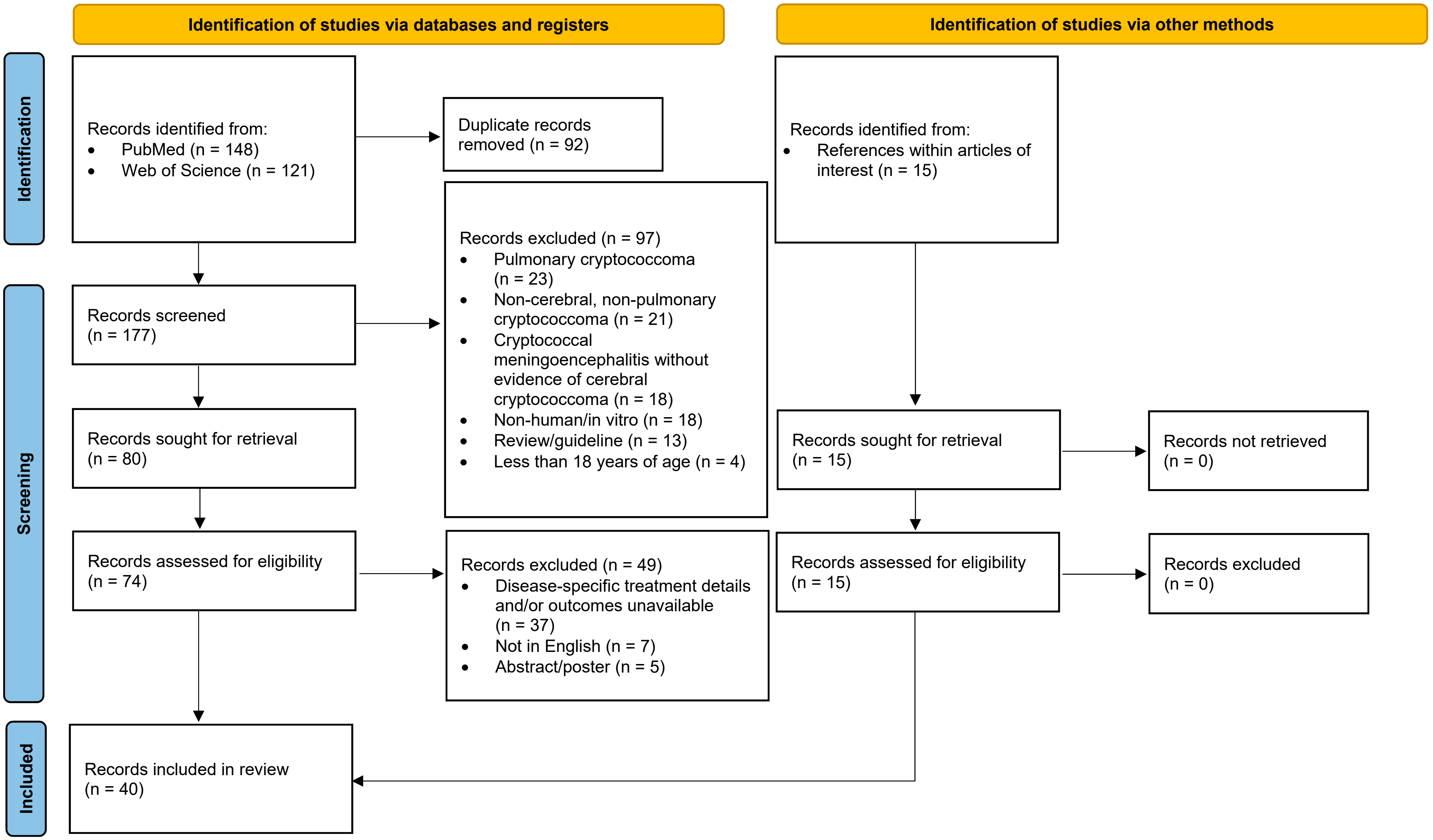



Page MJ, et al., The PRISMA 2020 statement: an updated guideline for reporting systematic reviews. BMJ, 2021. **372**: p. n71.
